# The Dental Laboratory

**Published:** 1900-04-15

**Authors:** J. E. Nyman


### THE DENTAL LABORATORY.

Those which are only a fenced-off quarter section of the operating room, usually look more like an enlarged waste paper basket than anything else, and it is no wonder that a mediocre class of work emenates from such a place. The dentist only goes into it, as a rule, when he must of necessity, and emerges from it as speedily as possible. Such laboratories are usually covered with dust from ceiling to floor, with an excess in the corners, and scattered about on the benches and shelves are hideous models of plaster teeth, and I can easily imagine one who is forced to spend an hour or so at the close of a hard day in such a laboratory, taking to drink, if he is a drinking man.

At the time I remodeled my offices, the question arose as to whether it would be best to fence off a quarter section, for a laboratory, or not, I finally made up my mind not to do so, but had a special roll-topped desk constructed, which I knew I would have to keep clean, and so instead of having two small stuffy rooms which were difficult to ventilate, I have one large room in which it is a pleasure to operate. I find that by arranging things systematically I can keep everything necessary for my prosthetic work just as conveniently in that desk as I could if I had a laboratory, I have also schooled myself in the art of mixing plaster and running models, without bespattering everything within an area of six feet.—*Dr. J. E. Nyman, Dental Review, in March*, 1900.

